# Chained Activation of the Motor System during Language Understanding

**DOI:** 10.3389/fpsyg.2017.00199

**Published:** 2017-02-20

**Authors:** Barbara F. Marino, Anna M. Borghi, Giovanni Buccino, Lucia Riggio

**Affiliations:** ^1^Dipartimento di Psicologia, Università di Milano-BicoccaMilano, Italy; ^2^Dipartimento di Psicologia, Università di BolognaBologna, Italy; ^3^National Research Council (CNR), Istituto di Scienze e Tecnologie della CognizioneRome, Italy; ^4^Dipartimento di Scienze Mediche e Chirurgiche, Università “Magna Graecia” di CatanzaroCatanzaro, Italy; ^5^Dipartimento di Neuroscienze, Sezione di Fisiologia, Università di ParmaParma, Italy

**Keywords:** embodied language, motor chains, motor system, affordances, reaction times

## Abstract

Two experiments were carried out to investigate whether and how one important characteristic of the motor system, that is its goal-directed organization in motor chains, is reflected in language processing. This possibility stems from the embodied theory of language, according to which the linguistic system re-uses the structures of the motor system. The participants were presented with nouns of common tools preceded by a pair of verbs expressing grasping or observational motor chains (i.e., grasp-to-move, grasp-to-use, look-at-to-grasp, and look-at-to-stare). They decided whether the tool mentioned in the sentence was the same as that displayed in a picture presented shortly after. A primacy of the grasp-to-use motor chain over the other motor chains in priming the participants' performance was observed in both the experiments. More interestingly, we found that the motor information evoked by the noun was modulated by the specific motor-chain expressed by the preceding verbs. Specifically, with the grasping chain aimed at using the tool, the functional motor information prevailed over the volumetric information, and *vice versa* with the grasping chain aimed at moving the tool (Experiment 2). Instead, the functional and volumetric information were balanced for those motor chains that comprise at least an observational act (Experiment 1). Overall our results are in keeping with the embodied theory of language and suggest that understanding sentences expressing an action directed toward a tool drives a chained activation of the motor system.

## Introduction

According to the theory of re-use (Anderson, 2010), evolution works in a conservative way, building on previously formed systems. In line with this general view, the embodied theory of language (Gallese and Lakoff, 2005; Glenberg, 2007; Gallese, 2008; Glenberg and Gallese, 2012) claims that the linguistic system re-uses the structures and the organization characterizing the motor system. From this perspective, language comprehension is rooted in action as it recruits the same neural areas that are active while performing movements.

Thus far, some of the most striking evidence for embodied language comes from psychophysiological and neuroimaging studies documenting an activation of the motor system during the comprehension of nouns referring to manipulable objects (i.e., tools), which parallels the activation of the same system while both actively manipulating and passively viewing these objects (see e.g., Martin et al., 1996; Grafton et al., 1997; Binkofski et al., 1999; Chao and Martin, 2000; Gerlach et al., 2002; Creem-Regehr and Lee, 2005; for a review see Martin, 2007). For example, Cattaneo et al. (2010), using transcranial magnetic stimulation (TMS) technique, found an involvement of ventral premotor cortex in processing of nouns referring to tools. Rueschemeyer et al. (2010) with functional magnetic resonance imaging (fMRI) showed that functionally manipulable words (i.e., nouns denoting man-made objects that require manipulation for use, such as “hammer”) elicit greater levels of activation in the fronto-parietal sensorimotor areas than volumetrically manipulable words (i.e., nouns denoting man-made objects that can be held in the hand but function without regular manipulation, such as “clock”). Similar findings were obtained in a TMS study by Gough et al. (2012).

Coupled evidence for an activation of motor system while processing of nouns referring to manipulable objects has been collected also in behavioral studies such as those investigating the influence of tool noun presentation on planning and executing hand movements in categorization tasks (Tucker and Ellis, 2004), lexical decision tasks (Myung et al., 2006), and gesture imitation tasks (Bub et al., 2008). For example, it has been demonstrated that planning reach-to-grasp movements aimed at using a tool interacts with the semantic activation of nouns related to the action goal of the tool use (Lindemann et al., 2006). More recently, it has been shown that the motor activation by nouns referring to manipulable objects is time-locked to the very moment at which the meaning of these nouns is accessed (Marino et al., 2013) and overlaps with the one driven by passive viewing of the manipulable objects to which the nouns are related (Marino et al., 2014).

Although, the evidence of an activation of the motor system by action-related nouns is compelling, little focus has been placed upon the aspects of this activation. The embodied theory of language predicts that the basic features characterizing the motor system should be maintained in processing action-related language. The present study is addressed at investigating this possibility. Specifically, it is aimed at testing whether and how one important characteristic of the motor system, that is its goal-directed organization in motor chains, is reflected in language processing.

The chained organization of the motor system has been recently described in some neurophysiological studies on monkeys (e.g., Fogassi et al., 2005; Bonini et al., 2011). These studies showed that, in the parietal and premotor cortices, the majority of neurons coding a specific motor act have a different activation pattern depending on the overall goal of the action sequence in which the act is embedded. For example, among the neurons selective for object grasping, some discharge best when the act is executed for placing the object into a container while others for eating it. These results show that a basic mechanism of the motor system is structuring of the same motor act in different motor chains. In humans, the chained organization of the motor system has been revealed by the results collected in a brain imaging study by Iacoboni et al. (2005) who found a significant signal increase in the brain areas where hand actions are represented (the posterior part of the inferior frontal gyrus and the adjacent sector of the ventral premotor cortex) while watching a grasping gesture embedded in a motor chain (i.e., grasp-to-drink and grasp-to-clean) as compared to watching the same gesture alone. An important assumption of the present study is that such chained organization can characterize language as well.

A model based on motor chains and language has been proposed by Chersi et al. (2010) to account for contradictory findings that understanding verbs associated to different effectors can lead to either facilitation or interference in motor responses (e.g., Boulenger et al., 2006). However, to our knowledge, the issue of a chained activation of the motor system during the comprehension of linguistic material related to action has not yet been directly addressed by either behavioral or brain imaging studies. To investigate this possibility we used the procedure employed by Stanfield and Zwaan (2001), and by Borghi and Riggio (2009). Participants were presented with sentences composed by the noun of a tool and a pair of verbs expressing different grasping motor chains (i.e., grasp-to-move the tool, grasp-to-use the tool) or observational motor chains (look at-to-grasp the tool, look at to stare the tool). Each sentence was followed by a picture of a tool graspable with either a precision or power grip, with its handle oriented to the right and its functional part (i.e., the tip) oriented to the left, and *vice versa*. Participants were required to decide whether the tool in the picture was the same as the one presented in the sentence (i.e., word-picture matching task). If the chain organization of motor system is encoded in language, then the priming effect typically observed when the object described in the sentence overlaps with the object represented in the following picture, should be shaped by the motor chain expressed by the pair of verbs with which the noun was combined. In particular, given that objects are represented primarily in terms of their actions, a priming advantage of the motor chains containing the act of grasping over the pure observational motor chain should be observed. Moreover, since tools are represented primarily in terms of their function (e.g., Costantini et al., 2011), a primacy of the grasp-to-use motor chain over the other motor chains containing the act of grasping should be found. Finally, if a chained activation of the motor system during language processing occurs, then the motor information evoked by the noun of the tool should contain different details depending on the motor chain expressed by the sentence in which the noun is embedded. Specifically, details related to the relation between the hand and the graspable part of the tool (i.e., the handle) should be evoked by nouns of tools embedded in the grasp-to-move and look at-to-grasp motor chains. In contrast, details pertaining also to the relation between the hand and the functional part of the tool (i.e., the tip) should be somehow evoked by nouns of tools embedded in the grasp-to-use motor chain.

## Experiment 1

### Materials and methods

#### Participants

Thirty-four students of the University of Parma (14 males and 20 females, mean age ± SD, 21.2 ± 3.8 years) took part in the experiment. All were right-handed native Italian speakers (mean Edinburgh Handedness Questionnaire score ± SD, 0.85 ± 0.13, Oldfield, 1971) and were unaware of the purpose of the study. The participants had normal or corrected to normal vision and reported no history of speaking and/or motor disorders. All the participants gave a written informed consent before testing. The study was conducted in accordance with the ethical standards laid down in the 1964 Declaration of Helsinki and fulfilled the ethical standard guidelines recommended by the Italian Association of Psychology. The experimental protocol was also approved by the research ethics committee at the University of Parma.

#### Materials

Fourteen digital color photographs of common tools were selected. All the tools, which had a shape elongated along the vertical axis, were composed of two structurally separated parts, the handle and the tip serving to accomplish the tool function. All the tool exemplars were chosen so that neither their handle not their tip differently popped out of the background due to a large difference in color contrast. Half of the tools were graspable with a power grip (e.g., hammer), while the other half were graspable with a precision grip (e.g., pen). A complete list of the tools is provided in Table 1. Each tool was scaled to be displayed within a 130 × 130 mm white frame, with its handle pointing downwards both to the left and to the right, and its tip pointing upwards both to the right and to the left, respectively.

**Table 1:** **Tools used in Experiment 1 and 2**.

**Precision grip**		**Power grip**	
Cucchiaio	(*spoon*)	Pennello	(*paintbrush*)
Chiave	(*key*)	Martello	(*hammer*)
Penna	(*pen*)	Pinze	(*pliers*)
Pennellino	(*little paintbrush*)	Spazzola	(*hairbrush*)
Forchetta	(*fork*)	Schiaccianoci	(*nutcracker*)
Matita	(*pencil*)	Cacciavite	(*screwdriver*)
Fiammifero	(*match*)	Pettine	(*comb*)

We also created 6 different kinds of Italian imperative sentences in which a pair of transitive verbs, separated by the copulative conjunction “e” (English translation: *and*), was followed by a determinative article and a noun that referred to one of the tools represented in the pictures (i.e., verb 1 + conjunction + verb 2 + determinative article + noun). The sentences could include two action verbs (i.e., “afferra e sposta”—*grasp and move*, or “afferra e usa”—*grasp and use*), an observational verb and an action verb (i.e., “osserva e prendi”—*look at and grasp*, “osserva e indica”—*look at and point*, or “afferra e fissa”—*grasp and stare*), or two observational verbs (i.e., “osserva e fissa”—*look at and stare*). Sentences that included the verbal pairs “look at and point” or “grasp and stare” served as catch trials to induce the participants to process the whole sentence (see below). All the verb pairs included in the critical sentences were composed of 6 syllables, and had a similar additive relative lexical frequency (“afferra e sposta” = 1.70 + 20.49 = 22.19, “afferra e usa” = 1.70 + 28.63 = 30.33, “osserva e prendi” = 22.86 + 6.66 = 29.52, “osserva e fissa” = 22.86 + 9.90 = 32.76 in occurrences per million; see (Laudanna et al., 1995) ~ 3,798,000 words). The nouns referring to the tools graspable with either a precision or a power grip were matched for syllable number [average values: 3.00 vs. 3.14 syllables, *t*_(1, 12)_ = 0.36, *p* = 0.73] and lexical frequency [average values: 10.59 vs. 1.78 in occurrence per million, *t*_(1, 12)_ = 1.46, *p* = 0.17].

#### Procedure

The experiment was carried out in a sound-attenuated room, dimly illuminated by a halogen lamp directed toward the ceiling. The participants, tested individually, sat comfortably in front of the screen of a computer monitor (a ViewSonic 18 inch flat color CRT monitor with a 1024 × 768 pixel resolution, interfaced with an Intel R Core™ 2.40 GHz computer equipped with an ATI Radeon HD 2600 Pro Video Board) with their head supported by a chin rest in order to maintain a stable eye-to-screen distance of 57 cm.

**Figure 1:**
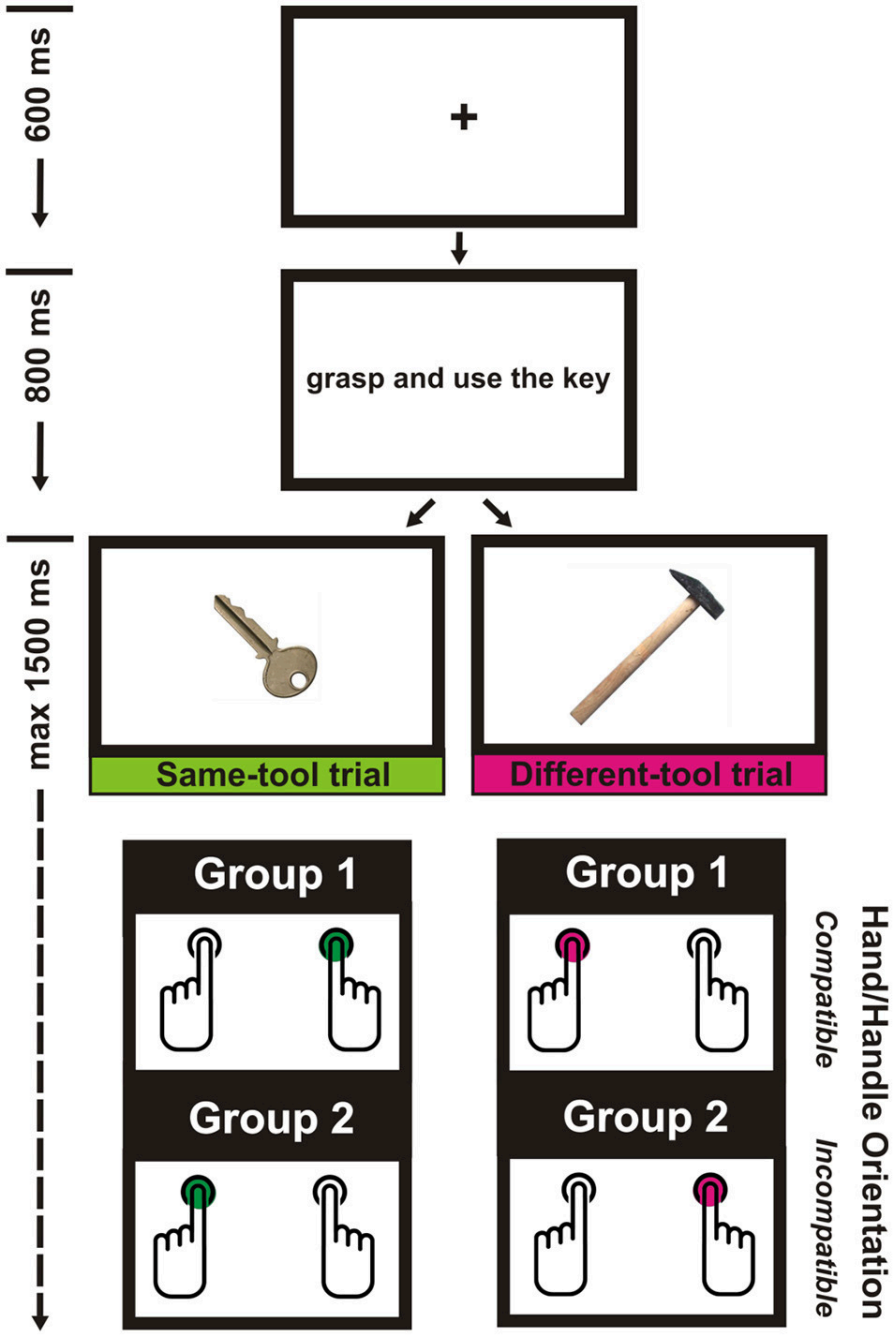
**The experimental procedure**.

Each trial started with a black fixation cross displayed at the center of a white background. After a delay of 600 ms, the fixation cross was replaced by a sentence. The sentence was centrally displayed and written in black lowercase Courier New bold font (point size = 24). The sentence remained visible for 800 ms. After the offset of the sentence a digital photograph of a tool centrally appeared. The timer started operating simultaneously to the onset of the visual tool which remained visible until the participant responded, or until 1500 ms had passed (see Figure 1).

The participants were randomly assigned to one of two groups. Those in the first group were asked to press the “p” key with their right index finger when the tool in the photograph was the same as that mentioned in the sentence, and to press the “q” key with their left index finger when it was not; the participants in the other group were required to do the opposite. All the participants were instructed to refrain from responding in case the sentence included the verb pair “look at and point” or “grasp and stare” (i.e., catch-trials). The keyboard was positioned in front of the participants, so that the two response keys were placed symmetrically with respect to the participants' body midline. All the participants were informed that their response times (RTs) would be recorded and were invited to respond as quickly as possible while still maintaining accuracy. They received feedback after pressing the wrong key in a critical trial (“ERROR”), after pressing a key in a catch trial (“ERROR”), after taking more than 1500 ms to respond (“TOO SLOW”), or after responding correctly (“CORRECT”). The feedback remained visible for 1500 ms.

In the first phase of the experimental session, the participants performed a block of 48 practice trials. Different tools as those used in the following test phase were used. If the participants felt confident with the task, then the test phase was started, otherwise another block of practice trials was run. In the test phase, the participants performed a block of 336 trials. The order of stimulus presentation was randomized and Verb Pair (“grasp and move,” “grasp and use,” “look at and grasp,” “look at and point,” “look at and stare,” “grasp and stare”), Tool Handle Orientation (left, right), and Tool Grip (precision, power) factors were fully balanced. Each of the 6 verb pairs was combined with each of the 14 tool nouns (for a total of 84 different sentences). Each sentence, which was presented 4 times during the whole test phase, was followed twice by the photograph of the tool mentioned in the sentence (i.e., same-tool trials),—with the handle directed downward once to the left and once to the right—, and twice by the photograph of a tool not mentioned in the sentence (i.e., different-tool trials),—with the handle directed downward once to the left and once to the right. The photos used in the different-tool trials depicted tools which were graspable with the same kind of grip as that required to grasp the tool mentioned in the sentence for half of the times, and with a different kind of grip for the other half of the times.

Throughout the test phase, the participants could take a break after every 42 trials. For each trial, RTs and errors were recorded. Stimulus presentation and response collection were controlled using the software package E-Prime, version 1.1. (Psychology Software Tools, Inc.).

### Results

**Figure 2:**
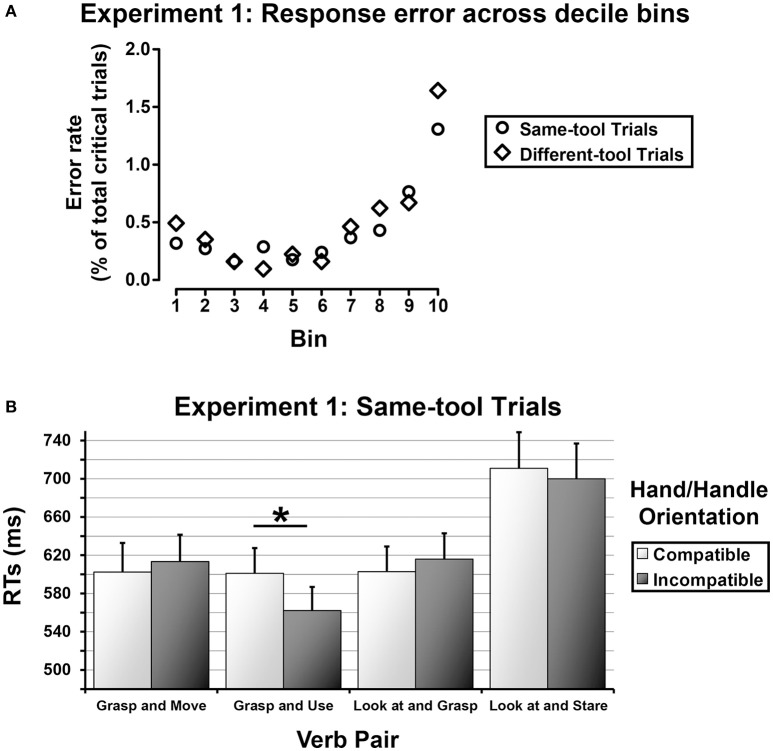
**(A)** Experiment 1—Response errors over time. The critical trials were sorted according to increasing RTs and grouped into 10 different temporal bins of approximately 285 trials each. For each bin, the averaged error rate was computed separately for the same-tool trials (outline disks) and the different-tool trials (outline diamonds). **(B)** Experiment 1—Same-tool trials. Averaged RTs as a function of Verb Pair separately for compatible and incompatible Hand/Handle Orientations (light gray rectangles and dark gray rectangles, respectively). The error bars represent the standard error.

All cases in which the participants responded to a critical trial by pressing a wrong key and all cases in which the participants responded to a catch trial were considered as errors (i.e., response errors and catch errors, respectively). The grand mean percentage error was 16.92% (response errors = 10.72%, catch errors = 6.20%). The catch trials and the wrong critical trials were excluded from the analysis. Six participants (2 from the first group and 4 from the second group) were removed from the analysis because their error rate was statistical outlier (i.e., two standard deviation higher than the error rate grand mean). Before being analyzed, the response times (RTs) measured for the correct critical trials were screened for outliers: RTs two standard deviations higher or lower than the individual grand mean were omitted from the analysis (19.23%). Given that there was no speed-accuracy tradeoff, as determined by plotting the error rate across decile temporal bins (see Figure 2A), the remaining RTs measured for the same-tool trials and for the different-tool trials were separately submitted to a mixed analysis of variance (ANOVA) with Response Hand (left, right) as a between-subjects factor and Tool Grip (power, precision), Verb Pair (“grasp and move,” “grasp and use,” “look at and grasp,” “look at and stare”), and Hand/Handle Orientation (compatible, incompatible) as within-subjects factors. This latter factor was obtained by combining the orientation of the handle of the tool shown in photographs (left, right) with the hand used to give the response (left, right). In the ANOVA on RTs measured for the different-tool trials, Grip Congruency (congruent, incongruent) between the tool mentioned in the sentence and the visual tool was considered as an additional within-subjects factor. Besides, the levels of Tool Grip factor referred to the hand posture appropriate for grasping the visual tool. In both the ANOVAs, skewness and kurtosis of RTs distributions were examined to determine whether the data were normally distributed. Following West et al. (1995), we assumed as reference to substantial departure from normality, an absolute skewness index > 2 and an absolute kurtosis index > 7. Violations of sphericity were controlled using Mauchly's test of sphericity and either Greenhouse-Geisser or Huynh-Feldt corrections were applied according to Girden (1992). If a Greenhouse-Geisser epsilon of >0.75 was found, the Huynth-Feldt corrected value was used for that parameter. Otherwise the Greenhouse-Geisser corrected value was used. Partial eta squared values ($\eta_{p}^{2}$) were reported as a metric of effect size for all significant ANOVA contrasts. The Duncan's test was used for post-hoc comparisons with a significance level set at 0.05. Only the significant results will be reported.

#### Same-tool trials

RTs were normally distributed as the skewness and kurtosis indexes were within acceptable ranges of normality (*skewness* = 0.93 ± 0.05; *kurtosis* = 0.38 ± 0.10). The ANOVA revealed a main effect of Tool Grip [*F*_(1, 26)_ = 18.37, *MSE* = 155,733, *p* < 0.001, $\eta_{p}^{2}$ = 0.41], indicating longer response latencies when the tool was graspable with a power grip than with a precision grip (647 vs. 609 ms). The analysis also showed a main effect of Verb Pair [*F*_(1.9, 49.46)_ = 36.83, *MSE* = 540959, *p* < 0.0001, $\eta_{p}^{2}$ = 0.59]. *Post-hoc* comparisons indicated that RTs were faster for the sentences comprising the verb pair “grasp and use” (582 ms) as compared to all the other verb pairs (“grasp and move” = 608 ms, *p* < 0.05; “look at and grasp” = 609 ms, *p* < 0.05; “look at and stare” = 705 ms, *p* < 0.001). RTs measured for the verb pairs “grasp and move” and “look at and grasp” did not differ from each other (*p* = 0.91), but they were significantly faster than RTs measured for the verb pair “look at and stare” (all *ps* < 0.001). In addition, there was a significant 2-ways interaction between Verb Pair and Hand/Handle Orientation [*F*_(3, 78)_ = 2.80, *MSE* = 17675, *p* < 0.05, $\eta_{p}^{2}$ = 0.10], indicating that in the “grasp and use” trials the participants were faster when their response hand was spatially incompatible with the tool handle (562 ms) than when the response hand and the tool handle were spatially compatible (601 ms, *p* < 0.02, see Figure 2B). No effect of Hand/Handle Orientation was found for the other verb pairs.

#### Different-tool trials

RTs were normally distributed as the skewness and kurtosis indexes were within acceptable ranges of normality (*skewness* = −0.33 ± 0.05; *kurtosis* = 0.02 ± 0.10). The ANOVA revealed a main effect of Verb Pair [*F*_(3, 60)_ = 20.34, *MSE* = 346,782, *p* < 0.0001, $\eta_{p}^{2}$ = 0.50], with longer response latencies for the sentences comprising the verbal pair “look at and stare” (715 ms) as compared to the other sentences (all *p*s < 0.001). In contrast with the results found for the same-tool trials, the *post-hoc* comparisons revealed no significant differences between the sentences comprising the verb pair “grasp and use” (628 ms) and those expressing the other grasping sequences (“grasp and move” = 619 ms, “look at and grasp” = 654 ms, all *p*s > 0.07).

### Discussion

The results of both the analyses showed converging support for our hypothesis that the chained organization is encoded in language. In line with the idea that objects are represented primarily in terms of the actions they afford, we found that in the same-tool trials the sentences expressing the pure observational motor chain (i.e., look-at-to-stare) primed the participants' responses less efficiently than the sentences expressing a chain in which the motor act of grasping was embedded (for convergent findings on the difference between observation and action sentences, see Borghi and Riggio, 2009; Costantini et al., 2011). In addition, consistently with the fact that tools are represented primarily in terms of their function, the time required to perform the word-picture matching task in the same-tool trials was modulated by the final goal of the action expressed by the sentence. The sentences expressing the grasp-to-use motor chain primed the participants' responses more efficiently than the sentences expressing the grasping motor chains not overtly aimed at using the tool (i.e., grasp-to-move and look-at-to-grasp). This finding (see Costantini et al., 2011; Lee et al., 2012, for similar results) points toward the possibility that the grasping motor chain aimed at using a tool, as compared with the other motor chains, selectively triggers the activation of the most crucial motor information of the conceptual representation of the tool, that is its functional information (i.e., the motor information about how to use an object). As already observed in previous studies, functional knowledge associated with manipulable man-made objects is an important component of their conceptual representation (e.g., Kellenbach et al., 2003; Vainio et al., 2008; Jax and Buxbaum, 2010). This knowledge is activated very early when identifying words or reading them for meaning and showed a marked benefit in priming tasks (Moss et al., 1997; Myung et al., 2006; Bub et al., 2008; Bub and Masson, 2010).

The idea that the grasp-to-use motor chain drove a selective activation of the functional information during tool noun understanding is further supported by the third result collected in the same-tool trials. In particular, we found that the grasp-to-use motor chain led to faster responses when the response hand and the handle of the visually presented tool were spatially incompatible. While the inversion of the affordance effect has been occasionally found in previous studies with objects (e.g. Pellicano et al., 2010; Kostov and Janyan, 2015), to our knowledge it is the first time in which it is reported in language processing. This remarkable inversion of the classic affordance effect (i.e., faster responses when the response hand and the handle of a graspable object are spatially compatible, see e.g., Tucker and Ellis, 1998) suggests that the functional information evoked by the grasp-to-use motor chain included details pertaining to the tool tip which subserves tool usage. It is likely that the activation of these details drove the attention of the participants to focus on the functional portion of the tool during the successive processing of the visual stimulus, so that the tip of the tool acquired a directional meaning and generated the inverted affordance effect which parallels the Simon effect that occurs with centrally presented stimuli conveying spatial information, such as arrows (e.g., Tipples, 2002). Notably, recent findings suggest that the way we shift attention or explore an object is biased toward action-relevant information (e.g., Handy et al., 2003; Roberts and Humphreys, 2011; Ambrosini and Costantini, 2017). In particular, Ambrosini and Costantini (2017) showed that participants mostly fixate the action-related, functional part of the tools, regardless of its visual saliency. Crucially, the effect was strongly reduced when participants were required to tie their hands behind their back. The results lead to the conclusion that the action-relevant object information at least in part guides gaze behavior and visual attention.

The lack of a canonical affordance effect in the sentences expressing the grasping motor chains not overtly aimed at tool usage does not imply that no motor information was evoked during the comprehension of the tool noun. Indeed, these sentences were significantly more effective than the sentences containing the pure observational motor chain in priming participants' responses. More likely, the non-functional grasping chains triggered, along with the functional information, the activation of the volumetric information (i.e., the motor information about how to manipulate an object) with the result that the details associated to the tool tip were contrasted with the details associated to the tool handle, generating no observable bias in the participants' responses. This is in keeping with the results of recent studies (Bub et al., 2008; Bub and Masson, 2010; Jax and Buxbaum, 2010; Pellicano et al., 2010) showing that both functional and volumetric information are activated in parallel by visual tools and that a convergence between these two kinds of information takes place, with the result of a conflict or a summation depending on their mutual consistency.

The idea of a parallel and converging activation of functional and manipulation information during tool noun comprehension is also supported by the results of the different-tool trials. In particular, when the tool in the sentence and the tool in the picture did not share the same function, the advantage for the grasp-to-use motor chain over the other grasping motor chains disappeared. This finding converges with results collected by Myung et al. (2006), showing that nouns denoting man-made objects can prime one another only if hand gestures related to their conventional use are similar (e.g., running a lexical decision on the word “piano” was faster when it was preceded by the word “typewriter” than by a control word), and indicates that presenting the picture of a different tool as that mentioned in a grasp-to-use sentence, suppressed the primacy of the functional information evoked by the noun, thus redressing the balance between the functional and manipulation information.

## Experiment 2

The word-picture matching task used in Experiment 1 turned out to be quite difficult. Indeed, for most of the participants more than one block of training trials was required. Moreover, at the end of the experimental session, the participants often reported that the task was quite demanding, mainly because of the high number of verbs and their combinations they had to retain in order to accomplish the task. The difficulty in performing the word-picture matching task was also revealed by the grand mean percentage error which resulted to be relatively high (16.92% of total trials) and by the high variability of the data. In Experiment 2, we aimed at reducing the task difficulty by decreasing the number of the verb pairs used in both critical and catch trials. We also aimed at reproducing the inverted affordance effect observed in Experiment 1.

### Materials and methods

#### Participants

Twenty-four right-handed students of the University of Parma (12 males and 12 females, mean age ± SD, 26.25 ± 3.47 years, mean Edinburgh Handedness Questionnaire score ± SD, 0.75 ± 0.19) took part in the experiment. The selection procedure of the participants was the same as in Experiment 1. All the participants gave a written informed consent before testing. The study was conducted in accordance with the ethical standards laid down in the 1964 Declaration of Helsinki and fulfilled the ethical standard guidelines recommended by the Italian Association of Psychology. The experimental protocol was also approved by the research ethics committee at the University of Parma.

#### Materials

Fourteen digital color photographs of tools, identical to those in Experiment 1, were used as visual stimuli. Unlike Experiment 1, in this experiment we created only 4 kinds of imperative sentences with the same structure as the one previously used. These sentences always included the verb “afferra” (or its synonymous “prendi,” English translation: *grasp*) along with an action verb (i.e., “afferra e usa”—*grasp and use*, “prendi e muovi”—*grasp and move*, “afferra e disturba”—*grasp and disturb*, and “prendi e ostacola”—*grasp and hinder*). The sentences comprising the verbal pairs “grasp and use” and “grasp and move” were already used in Experiment 1 and served as critical trials. Sentences comprising the verbal pairs “grasp and disturb” and “grasp and hinder” worked as catch trials. It should be noted that the combination of these two verb pairs with a noun referring to a tool gave rise to sentences which did not make sense. We used this kind of sentences as catch trials so that the participants would not have to memorize the verb pairs to which they have to refrain from responding. As in Experiment 1, the verb pairs used in the critical sentences were composed of 6 syllables, and had a similar additive relative lexical frequency. Moreover, nouns referring to the precision-grip and power-grip tools were matched for syllable number and lexical frequency (see Materials of Experiment 1).

#### Procedure

The same procedure was used as in Experiment 1, except that the participants performed a block of 32 trials in the training phase and a block of 224 trials in the test phase. In both phases, the order of stimulus presentation was randomized and Verb Pair (“grasp and use,” “grasp and move,” “grasp and disturb,” and “grasp and hinder”), Tool Handle Orientation (left, right), and Tool Grip (precision, power) factors were fully balanced. As in the test phase of Experiment 1, each of the 4 verb pairs was combined with each of the 14 tool nouns (for a total of 56 different sentences). Each sentence, which was presented 4 times during the whole test phase, was followed twice by the photograph of the tool mentioned in the sentence (i.e., same-tool trials),—with the handle pointing once downward to the left and once downward to the right—, and twice by the photograph of a tool not mentioned in the sentence (i.e., different-tool trials),—with the handle pointing once downward to the left and once downward to the right. The photographs used in the different-tool trials depicted tools which were graspable with the same kind of grip as that required to grasp the tool mentioned in the sentence for half of the times, and with a different kind of grip for the other half of the times. Throughout the test phase, the participants could take a break after every 56 trials.

### Results

**Figure 3:**
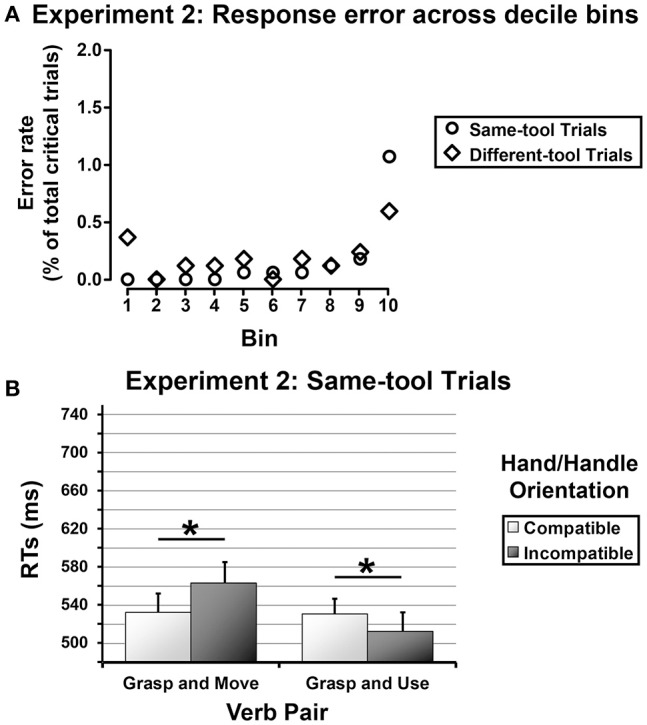
**(A)** Experiment 2—Error rate across decile temporal bins of approximately 108 trials each, for the same-tool trials (outline disks) and the different-tool trials (outline diamonds), separately. **(B)** Experiment 2—Same-tool trials. Mean RTs as a function of Verb Pair separately for congruent and incongruent Hand/Handle Orientations (gray squares and black squares, respectively). The error bars represent the standard error.

The grand mean percentage error was 3.16% of total trials (response errors = 1.96%, catch errors = 1.17%). The catch trials and the wrong critical trials were excluded from the analysis. Three participants were removed from the analysis because their error rate was statistical outlier (i.e., two standard deviation higher than the error rate grand mean). Before being analyzed, the RTs measured for the correct critical trials were screened for outliers according to the same criteria as applied in Experiment 1 (4.25% of correct RTs were omitted from the analysis). Given that there was no speed-for-accuracy tradeoff, as determined by a plot of error rate across decile temporal bins (see Figure 3A), the remaining RTs measured for the same-tool trials and for the different-tool trials were separately submitted to a mixed ANOVA with the same between- and within-subjects factors as considered in Experiment 1. As in Experiment 1, skewness and kurtosis of RTs distributions were examined to determine whether the data were normally distributed (values for departure from normality: skewness >2 and kurtosis >7; West et al., 1995). Partial eta squared values ($\eta_{p}^{2}$) were reported as a metric of effect size for all significant ANOVA contrasts. The Duncan's test was used as a post-hoc test with a significance level set at 0.05. As before, only significant results have been reported.

#### Same-tool trials

RTs were normally distributed as the skewness and kurtosis indexes were within acceptable ranges of normality (*skewness* = 1.42 ± 0.07; *kurtosis* = 2.38 ± 0.14). As in Experiment 1, the ANOVA revealed a main effect of Tool Grip [*F*_(1, 20)_ = 29.76, *MSE* = 44576, *p* < 0.001, $\eta_{p}^{2}$ = 0.60], with longer response latencies when the tool was graspable with a power grip than with a precision grip (551 vs. 519 ms), as well as a main effect of Verb Pair [*F*_(1, 20)_ = 12.10, *MSE* = 28,776, *p* < 0.003, $\eta_{p}^{2}$ = 0.38], indicating faster RTs for sentences comprising the verbs “grasp and use” (522 ms) than “grasp and move” (548 ms). In addition, the ANOVA revealed a significant interaction between Tool Grip and Verb Pair [*F*_(1, 20)_ = 4.90, *MSE* = 6343, *p* < 0.04, $\eta_{p}^{2}$ = 0.20], indicating that the primacy of the “grasp-and use” sentences in priming subjects' responses was confined to the tools graspable with a precision grip (precision-grip tools = 499 ms, power-grip tools = 544 ms; *p* < 0.001). Noticeably, there was also a significant interaction between Verb Pair and Hand/Handle Orientation [*F*_(1, 20)_ = 15.67, *MSE* = 25,068, *p* < 0.001, $\eta_{p}^{2}$ = 0.44], showing an inversion of the affordance effect for the sentences containing the verbs “grasp and use” (congruent orientation = 531 ms, incongruent orientation = 513 ms; *p* < 0.05) and a classic affordance effect for the sentences containing the verbs “grasp and move” (congruent orientation = 533 ms, incongruent orientation = 563 ms; *p* < 0.003; see Figure 3B).

#### Different-tool trials

RTs were normally distributed as the skewness and kurtosis indexes were within acceptable ranges of normality (*skewness* = 1.72 ± 0.07; *kurtosis* = 3.47 ± 0.15). The ANOVA revealed a main effect of Hand/Handle Orientation [*F*_(1, 20)_ = 4.64, *MSE* = 13,117, *p* < 0.05, $\eta_{p}^{2}=$ 0.19], indicating faster RTs when subjects' response hand was spatially incompatible with the tool handle (590 ms) that when it was compatible (603 ms).

### Discussion

Decreasing the number of verb pairs used in the critical trials and reducing the cognitive load for the catch trials led to a marked reduction of the task difficulty. Indeed, the grand mean of percentage error was much lower than that of Experiment 1 (3.16 vs. 16.92%, respectively).

The results for the same-tool trials converge and extend those collected in the previous experiment. First, it is confirmed our interpretation that while understanding sentences obtained by combining a pair of action verbs with the noun of a tool, the motor information evoked by the noun is modulated by the specific motor-chain expressed by the verbs, as the primacy of the grasping-to-use motor chain over the grasp-to-move motor chain in priming word-picture matching was replicated.

Second, we were able to reproduce the inverted affordance effect observed in Experiment 1 for the “grasp and use” sentences. Interestingly, the removal of the sentences containing at least an observational act (i.e., “look at and grasp” and “look and stare”) enabled the classic affordance effect to be significantly detected in those trials where the grasp-to-move chain motor was used. It is likely that, under this condition, the non-functional grasping motor chain was plainly contrasted with the functional grasping motor chain, rather than being assimilated to the observational motor chains. As a consequence, the manipulation information evoked by the noun was capable of prevailing over the functional information, causing the affordance effect.

The results for the different-tool trials confirmed those collected in Experiment 1, since the advantage for the functional grasping motor chain over the non-functional motor chain in priming picture matching completely disappeared. In addition, there was an inversion of the affordance effect independently of the specific motor chain expressed by the sentence likely reflecting that coding the functional part of the visual tool assured a better recognition when it was inconsistent with the tool mentioned in the sentence.

## General discussion

In two experiments, we investigated the chained activation of the motor system during language understanding by exploring the priming effects exerted by the comprehension of a tool noun on the recognition of a tool displayed in a picture presented shortly after. The tool noun was combined with a pair of action verbs to form a sentence expressing different grasping and observational motor chains. Overall, our results reveal an important role of the context as defined by the sentence in affordance perception; this is in line with previous studies documenting the importance of both verbal and visual context in affordance perception (e.g., Kalénine et al., 2012, 2016; Borghi and Riggio, 2015). More specifically, we found that accessing the meaning of the tool noun activated motor information that changed in accordance with the final goal of the motor chain expressed by the verbs. The functional information prevailed on the volumetric information when the noun of the tool was embedded in the grasp-to-use motor chain. Motor information of the volumetric kind was likely activated for the grasp-to-move motor chain and when the tool noun was embedded in the look-at-to-grasp motor chain. Instead, action information was absent if the pure observational motor chain was used (i.e., look-at-and-stare), as the slower RTs reveal.

The advantage of the precision-grip tools over the power-grip tools that we found in both the experiments is in contrast with a number of studies showing lower RTs in responding to power-grip tools as compared to precision-grip tools (Ehrsson et al., 2001; Borghi and Riggio, 2009; Kalénine et al., 2014). It is likely that our functional motor chains selectively activate motor information related to a grasping gesture performed using a precision grip and this may be the likely reason why we found an advantage of the prevision-grip tool over the power-grip tool. This possibility is supported by the results of Experiment 2, showing that the precision-grip tool advantage is consistent only in trials where the grasp-to-use motor chain was used.

The current study is novel as it provides evidence that the motor information activated while understanding nouns of common tools has a goal-directed structure. In other words, the motor information these nouns are capable of activating is dependent on the aim of the global action performed with the tool and described by the other linguistic component of the sentence. This is in keeping with the idea that the activation of motor information by graspable objects always demands a selection of competing motor information (e.g., Fagg and Arbib, 1998). The context in which the objects are presented is thought to be a crucial factor that drives this selection. In an fMRI study, Iacoboni et al. (2005) showed reliable differences in the activity of the inferior frontal region during the observation of a grasping action (i.e., a hand seizing a cup) carried out in different contexts (i.e., “before tea” vs. “after tea”), likely indicating an automatic coding of the intention/goal behind the observed action (i.e., grasping the cup for drinking vs. grasping the cup for cleaning, respectively). More recently, Mizelle and Wheaton (2010), evaluating the neural correlates for tool identification and conceptual understanding with electroencephalography (EEG), found greater activity over the left temporo-parietal junction (that is thought to be part of a manipulation network) for tools presented in a matching functional-related context (i.e., followed by objects upon which the tools can act) than in a mismatching functional-related context. Preliminary evidence supporting a context-guided selection of motor information evoked by graspable objects was found also when a verbal presentation was used. For example, Lee et al. (2012), using eye-movement recording, found that the activation time course of functional and volumetric motor information by words of tools was modulated by the linguistic context in which the words are embedded (neutral vs. action-relevant context). Similarly, Marino et al. (2012) found that the motor information evoked during the comprehension of nouns of graspable objects was shaped by the sensorimotor specificity expressed by the sentence in which the nouns are embedded.

Since context (mostly expressed by verbs) and objects (expressed by nouns) are not presented in parallel by language, as it is for vision, it is an open question how these two factors interact in activating and selecting the motor information. Two processes are theoretically possible. According to Bub and Masson (2010), a process of activation-then-selection occurs during sentence understanding. Specifically, different types of motor information become active in response to a noun denoting a manipulable object. This activation is followed by a selection of relevant motor programs which is determined by the context expressed by the remaining linguistic components of the sentence. The current study provides evidence compatible with the alternative process, that is selection-then-activation: understanding action-related verbs, that in most of west European languages, such as Italian and English, precede the nouns defining the graspable objects to which the verbs refer, seems to automatically select a motor intention that enhances the activation by the nouns of the most contextually-relevant set of motor information among those possible.

Our finding of a goal-directed structure of motor information evoked by nouns referring to tools is consistent with the idea of a chained activation of the motor system during the comprehension of action-related linguistic material. This supports the embodied theory of language (Gallese and Lakoff, 2005; Glenberg, 2007; Gallese, 2008; Glenberg and Gallese, 2012) according to which, the linguistic system re-uses the structures of the motor system (see Rizzolatti and Arbib, 1998 for the basic role of the motor system in language evolution). Taken together the results of the current work suggest that processing combinations of action-related nouns and verbs involve the activation of the cortical motor system in a manner that parallels the organization of motor behavior and provide some hints that the syntax of language may be equivalent to the syntax of action (Dominey et al., 2003; Gallese, 2007, 2008; Clerget et al., 2009; Fazio et al., 2009; Pulvermüller and Fadiga, 2010; Marino et al., 2013).

## Author contributions

BM designed and performed the experiments, analyzed the data, contributed to the discussion of the data, wrote the manuscript, and prepared the figures. AB designed the experiments, prepared the stimuli, contributed to the discussion of the data, and revised the manuscript. GB designed the experiments, contributed to the discussion of the data, and revised the manuscript. LR designed the experiments, contributed to data analysis, contributed to the discussion of the data, and revised the manuscript. BM, AB, and LR reviewed the manuscript.

### Conflict of interest statement

The authors declare that the research was conducted in the absence of any commercial or financial relationships that could be construed as a potential conflict of interest.

## References

[B1] Ambrosini E. Costantini M. (2017). Body posture differentially impacts on visual attention towards tool, graspable and non-graspable objects. J. Exp. Psychol. Hum. Percept. Perform. 43, 360–370. 10.1037/xhp0000330 27831721

[B2] Anderson M. L. (2010). Neural reuse: a fundamental organizational principle of the brain. Behav. Brain Sci. 7, 245–266. 10.1017/S0140525X10000853 20964882

[B3] Binkofski F. Buccino G. Posse S. Seitz R. J. Rizzolatti G. Freund H. (1999). A fronto-parietal circuit for object manipulation in man: evidence from an fMRI-study. Eur. J. Neurosci. 11, 3276–3286. 10.1046/j.1460-9568.1999.00753.x 10510191

[B4] Bonini L. Serventi F. U. Simone L. Rozzi S. Ferrari P. F. Fogassi L. (2011). Grasping neurons of monkey parietal and premotor cortices encode action goals at distinct levels of abstraction during complex action sequences. J. Neurosci. 31, 5876–5886. 10.1523/JNEUROSCI.5186-10.2011 21490229 PMC6622840

[B5] Borghi A. M. Riggio L. (2009). Sentence comprehension and simulation of object temporary, canonical and stable affordances. Brain Res. 1253, 117–128. 10.1016/j.brainres.2008.11.064 19073156

[B6] Borghi A. M. Riggio L. (2015). Stable and variable affordances are both automatic and flexible. Front. Hum. Neurosci. 9:351. 10.3389/fnhum.2015.00351 26150778 PMC4473001

[B7] Boulenger V. Roy A. C. Paulignan Y. Deprez V. Jeannerod M. Nazir T. A. (2006). Cross-talk between language processes and overt motor behavior in the first 200 msec of processing. J. Cogn. Neurosci. 18, 1607–1615. 10.1162/jocn.2006.18.10.1607 17014366

[B8] Bub D. N. Masson M. E. (2010). On the nature of hand-action representations evoked during written sentence comprehension. Cognition 116, 394–408. 10.1016/j.cognition.2010.06.001 20579981

[B9] Bub D. N. Masson M. E. Cree G. S. (2008). Evocation of functional and volumetric gestural knowledge by objects and words. Cognition 106, 27–58. 10.1016/j.cognition.2006.12.010 17239839

[B10] Cattaneo Z. Devlin J. T. Salvini F. Vecchi T. Silvanto J. (2010). The causal role of category-specific neuronal representations in the left ventral premotor cortex (PMv) in semantic processing. Neuroimage 49, 2728–2734. 10.1016/j.neuroimage.2009.10.048 19853046

[B11] Chao L. L. Martin A. (2000). Representation of manipulable man-made objects in the dorsal stream. Neuroimage 12, 478–484. 10.1006/nimg.2000.0635 10988041

[B12] Chersi F. Thill S. Ziemke T. Borghi A. M. (2010). Sentence processing: linking language to motor chains. Front. Neurorobot. 4: pii: 4. 10.3389/fnbot.2010.00004 PMC2901116 20725506

[B13] Clerget E. Winderickx A. Fadiga L. Olivier E. (2009). Role of Broca's area in encoding sequential human actions: a virtual lesion study. Neuroreport 20, 1496–1499. 10.1097/WNR.0b013e3283329be8 19809371

[B14] Costantini M. Ambrosini E. Scorolli C. Borghi A. M. (2011). When objects are close to me: affordances in the peripersonal space. Psychon. Bull. Rev. 18, 302–308. 10.3758/s13423-011-0054-4 21327375

[B15] Creem-Regehr S. H. Lee J. N. (2005). Neural representations of graspable objects: are tools special? Cogn Brain Res. 22, 457–469. 10.1016/j.cogbrainres.2004.10.006 15722215

[B16] Dominey P. F. Hoen M. Blanc J. M. Lelekov-Boissard T. (2003). Neurological basis of language and sequential cognition: evidence from simulation, aphasia, and ERP studies. Brain Lang. 86, 207–225. 10.1016/S0093-934X(02)00529-1 12921765

[B17] Ehrsson H. H. Fagergren A. Forssberg H. (2001). Differential fronto-parietal activation depending on force used in a precision grip task: an fMRI study. J. Neurophysiol. 85, 2613–2623. 11387405 10.1152/jn.2001.85.6.2613

[B18] Fagg A. H. Arbib M. A. (1998). Modeling parietal-premotor interactions in primate control of grasping. Neural Netw. 11, 1277–1303. 10.1016/S0893-6080(98)00047-1 12662750

[B19] Fazio P. Cantagallo A. Craighero L. D'Ausilio A. Roy A. C. Pozzo T.. (2009). Encoding of human action in Broca's area. Brain 132(Pt 7), 1980–1988. 10.1093/brain/awp118 19443630

[B20] Fogassi L. Ferrari P. F. Gesierich B. Rozzi S. Chersi F. Rizzolatti G. (2005). Parietal lobe: from action organization to intention understanding. Science 308, 662–667. 10.1126/science.1106138 15860620

[B21] Gallese V. (2007). Before and below “theory of mind”: embodied simulation and the neural correlates of social cognition. Philos. Trans. R. Soc. Lond, B. Biol. Sci. 362, 659–669. 10.1098/rstb.2006.2002 17301027 PMC2346524

[B22] Gallese V. (2008). Mirror neurons and the social nature of language: the neural exploitation hypothesis. Soc. Neurosci. 3, 317–333. 10.1080/17470910701563608 18979384

[B23] Gallese V. Lakoff G. (2005). The Brain's concepts: the role of the Sensory-motor system in conceptual knowledge. Cogn. Neuropsychol. 22, 455–479. 10.1080/02643290442000310 21038261

[B24] Gerlach C. Law I. Paulson O. B. (2002). When action turns into words. Activation of motor-based knowledge during categorization of manipulable objects. J. Cogn. Neurosci. 14, 1230–1239. 10.1162/089892902760807221 12495528

[B25] Girden E. R. (1992). ANOVA: Repeated Measures. Newbury Park, CA: Sage Publications, Inc.

[B26] Glenberg A. M. (2007). Language and action: creating sensible combinations of ideas, in The Oxford Handbook of Psycholinguistics, ed Gaskell G. (Oxford: Oxford University Press), 361–370.

[B27] Glenberg A. M. Gallese V. (2012). Action-based language: a theory of language acquisition, comprehension, and production. Cortex 48, 905–922. 10.1016/j.cortex.2011.04.010 21601842

[B28] Gough P. M. Riggio L. Chersi F. Sato M. Fogassi L. Buccino G. (2012). Nouns referring to tools and natural objects differentially modulate the motor system. Neuropsychologia 50, 19–25. 10.1016/j.neuropsychologia.2011.10.017 22044649

[B29] Grafton S. T. Fadiga L. Arbib M. A. Rizzolatti G. (1997). Premotor cortex activation during observation and naming of familiar tools. Neuroimage 6, 231–236. 10.1006/nimg.1997.0293 9417966

[B30] Handy T. C. Grafton S. T. Shroff N. M. Ketay S. Gazzaniga M. S. (2003). Graspable objects grab attention when the potential for action is recognized. Nat. Neurosci. 6, 421–427. 10.1038/nn1031 12640459

[B31] Iacoboni M. Molnar-Szakacs I. Gallese V. Buccino G. Mazziotta J. C. Rizzolatti G. (2005). Grasping the intentions of others with one's own mirror neuron system. PLoS Biol. 3:e79. 10.1371/journal.pbio.0030079 15736981 PMC1044835

[B32] Jax S. A. Buxbaum L. J. (2010). Response interference between functional and structural actions linked to the same familiar object. Cognition 115, 350–355. 10.1016/j.cognition.2010.01.004 20156619 PMC2837086

[B33] Kalénine S. Mirman D. Middleton E. L. Buxbaum L. J. (2012). Temporal dynamics of activation of thematic and functional knowledge during conceptual processing of manipulable artifacts. J. Exp. Psychol. Learn. Mem. Cogn. 38, 1274–1295. 10.1037/a0027626 22449134 PMC3537173

[B34] Kalénine S. Shapiro A. D. Flumini A. Borghi A. M. Buxbaum L. J. (2014). Visual context modulates potentiation of grasp types during semantic object categorization. Psych. Bull. Rev. 21, 645–651. 10.3758/s13423-013-0536-7 24186270 PMC4008714

[B35] Kalénine S. Wamain Y. Decroix J. Coello Y. (2016). Conflict between object structural and functional affordances in peripersonal space. Cognition 155, 1–7. 10.1016/j.cognition.2016.06.006 27327864

[B36] Kellenbach M. L. Brett M. Patterson K. (2003). Actions speak louder than functions: the importance of manipulability and action in tool representation. J. Cogn. Neurosci. 15, 30–46. 10.1162/089892903321107800 12590841

[B37] Kostov K. Janyan A. (2015). Reversing the affordance effect: negative stimulus–response compatibility observed with images of graspable objects. Cognit. Process. 16(Suppl. 1), 287–291. 10.1007/s10339-015-0708-7 26233530

[B38] Laudanna A. Thorton A. Brown G. Burani C. Marconi L. (1995). Un corpus dell'italiano scritto contemporaneo dalla parte del ricevente, in III Giornate Internazionali di Analisi Statistica dei Dati Testuali, Vol. 1, eds Bolasco S. Lebart L. Salem A. (Roma: Cisu), 103–109.

[B39] Lee C. L. Middleton E. Mirman D. Kalénine S. Buxbaum L. J. (2012). Incidental and context-responsive activation of structure- and function-based action features during object identification. J. Exp. Psychol. Hum. Percept. Perform. 39, 257–270. 10.1037/a0027533 22390294 PMC3371276

[B40] Lindemann O. Stenneken P. van Schie H. T. Bekkering H. (2006). Semantic activation in action planning. J. Exp. Psychol. Hum. Percept. Perform. 32, 633–643. 10.1037/0096-1523.32.3.633 16822129

[B41] Marino B. F. Gallese V. Buccino G. Riggio L. (2012). Language sensorimotor specificity modulates the motor system. Cortex 48, 849–856. 10.1016/j.cortex.2010.12.003 21227411

[B42] Marino B. F. Gough P. M. Gallese V. Riggio L. Buccino G. (2013). How the motor system handles nouns: a behavioral study. Psychol. Res. 77, 64–73. 10.1007/s00426-011-0371-2 21879354

[B43] Marino B. F. Sirianni M. Volta R. D. Magliocco F. Silipo F. Quattrone A.. (2014). Viewing photos and reading nouns of natural graspable objects similarly modulate motor responses. Front. Hum. Neurosci. 8:968. 10.3389/fnhum.2014.00968 25538596 PMC4255516

[B44] Martin A. (2007). The representation of object concepts in the brain. Annu. Rev. Psychol. 58, 25–45. 10.1146/annurev.psych.57.102904.190143 16968210

[B45] Martin A. Wiggs C. L. Ungerleider L. G. Haxby J. V. (1996). Neural correlates of category-specific knowledge. Nature 379, 649–652. 10.1038/379649a0 8628399

[B46] Mizelle J. C. Wheaton L. A. (2010). Neural activation for conceptual identification of correct versus incorrect tool-object pairs. Brain Res. 1354, 100–112. 10.1016/j.brainres.2010.07.059 20701898

[B47] Moss H. E. McCormick S. F. Tyler L. K. (1997). The time course of activation of semantic information during spoken word recognition. Lang. Cogn. Proc. 12, 695–731. 10.1080/016909697386664

[B48] Myung J. Y. Blumstein S. E. Sedivy J. C. (2006). Playing on the typewriter, typing on the piano: manipulation knowledge of objects. Cognition 98, 223–243. 10.1016/j.cognition.2004.11.010 16399263

[B49] Oldfield R. C. (1971). The assessment and the analysis of handedness: the Edinburgh inventory. Neuropsychologia 9, 97–113. 10.1016/0028-3932(71)90067-4 5146491

[B50] Pellicano A. Iani C. Borghi A. M. Rubichi S. Nicoletti R. (2010). Simon-like and functional affordance effects with tools: the effects of object perceptual discrimination and object action state. Q. J. Exp. Psychol. 63, 2190–2201. 10.1080/17470218.2010.486903 20589580

[B51] Pulvermüller F. Fadiga L. (2010). Active perception: sensorimotor circuits as a cortical basis for language. Nat. Rev. Neurosci. 11, 351–360. 10.1038/nrn2811 20383203

[B52] Rizzolatti G. Arbib M. A. (1998). Language within our grasp. Trends Neurosci. 21, 188–194. 10.1016/S0166-2236(98)01260-0 9610880

[B53] Roberts K. L. Humphreys G. W. (2011). Action-related objects influence the distribution of visuospatial attention. Quart. J. Exp. Psychol. 64, 669–688. 10.1080/17470218.2010.520086 21113857

[B54] Rueschemeyer S. A. van Rooij D. Lindemann O. Willems R. M. Bekkering H. (2010). The function of words: distinct neural correlates for words denoting differently manipulable objects. J. Cogn. Neurosci. 22, 1844–1851. 10.1162/jocn.2009.21310 19583471

[B55] Stanfield R. A. Zwaan R. A. (2001). The effect of implied orientation derived from verbal context on picture recognition. Psychol. Sci. 12, 153–156. 10.1111/1467-9280.00326 11340925

[B56] Tipples J. (2002). Eye gaze is not unique: automatic orienting in response to uninformative arrows. Psychon. Bull. Rev. 9, 314–318. 10.3758/BF03196287 12120794

[B57] Tucker M. Ellis R. (1998). On the relations between seen objects and components of potential actions. J. Exp. Psychol. Hum. Percept. Perform. 24, 830–846. 10.1037/0096-1523.24.3.830 9627419

[B58] Tucker M. Ellis R. (2004). Action priming by briefly presented objects. Acta Psychol. (Amst.) 116, 185–203. 10.1016/j.actpsy.2004.01.004 15158182

[B59] Vainio L. Symes E. Ellis R. Tucker M. Ottoboni G. (2008). On the relations between action planning, object identification, and motor representations of observed actions and objects. Cognition 108, 444–465. 10.1016/j.cognition.2008.03.007 18452910

[B60] West S. G. Finch J. F. Curran P. J. (1995). Structural equation models with nonnormal variables: problems and remedies, in Structural Equation Modeling: Concepts, Issues, and Applications, ed Hoyle R. H. (Thousand Oaks, CA: Sage Publications, Inc), 56–75.

